# Variation in Both *IL28B* and *KIR2DS3* Genes Influence Pegylated Interferon and Ribavirin Hepatitis C Treatment Outcome in HIV-1 Co-Infection

**DOI:** 10.1371/journal.pone.0066831

**Published:** 2013-06-24

**Authors:** Ciara Keane, Daire O’Shea, Thomas Reiberger, Markus Peck-Radosavljevic, Gillian Farrell, Colm Bergin, Clair M. Gardiner

**Affiliations:** 1 Natural Killer Cell Group, School of Biochemistry & Immunology, Trinity College, Dublin, Ireland; 2 Department of GU Medicine & Infectious Diseases, St. James’s Hospital, Dublin, Ireland; 3 Division of Gastroenterology & Hepatology, Department of Internal Medicine III, Medical University of Vienna, Vienna, Austria; University of Sydney, Australia

## Abstract

Pegylated-IFN and ribavirin remains the current treatment for chronic HCV infection in patients co-infected with HIV-1, but this regimen has low efficacy rates, particularly for HCV genotype 1/4 infection, has severe side effects and is extremely costly. Therefore, accurate prediction of treatment response is urgently required. We have recently shown that the NK cell gene, *KIR2DS3* and a SNP associated with the *IL28B* gene synergise to increase the risk of chronic infection in primary HCV mono-infected patients. Identification of SNPs associated with the *IL28B* gene has also proven very powerful for predicting patient response to treatment. Patients co-infected with HIV-1 are of particular concern given they respond less well to HCV treatment, have more side effects and suffer a more rapid liver disease progression. In this study, we examined both *IL28B* and *KIR2DS3* for their ability to predict treatment response in a cohort of HIV-1/HCV co-infected patients attending two treatment centres in Europe. We found that variation in both host genetic risk factors, *IL28B* and *KIR2DS3*, was strongly associated with sustained virological response (SVR) to treatment in our co-infected cohort (n = 149). The majority of patients who achieved a rapid virological response (RVR) achieved a SVR. However, it is currently impossible to predict treatment outcome in patients who fail to achieve an RVR. In our cohort, the presence of host genetic risk factors, *IL28B-T* and *KIR2DS3* alleles, resulted in increased odds of treatment failure in these RVR negative patients (n = 88). Our data suggests that testing for host genetic factors will improve predicting treatment responsiveness in the clinical management of co-infected patients, and provides further evidence of the importance of the innate immune system in the immune response to HCV.

## Introduction

Significant advances have been made in the treatment of Hepatitis C virus (HCV) infection but challenges remain for patients that are co-infected with HCV and HIV-1 [1]. Co-infected patients have an accelerated progression of liver fibrosis [2], with consequent higher rates of cirrhosis, liver failure and hepatocellular carcinoma [3]. In addition sustained virologic response (SVR) rates to pegylated interferon-alpha (PEG-IFN) and ribavirin (RBV) are inferior when compared to HCV mono-infected patients with overall SVR rates of just 40–50% [3]. Presently PEG-IFN/RBV remains the standard of care for treatment of chronic HCV infection in co-infected patients, as direct acting antivirals (DAAs), such as telaprevir and boceprevir [4]–[6], are not yet approved for the treatment of co-infected individuals. To date there are few studies on the use of DAAs in co-infection, and their role and safe utilisation in this complex patient cohort requires further characterisation [7].

Given the high frequency of non-responsiveness to therapy and the toxicity and high cost of lengthy treatment schedules, identifying patients that are unlikely to achieve SVR to IFN-based treatment is extremely important from both patient care and economic stand-points [3]. Many host (age, liver fibrosis stage, pre-treatment IP10 levels) and viral factors (HCV genotype, baseline HCV-RNA levels) are known to influence likelihood of SVR. Additionally the ‘on-therapy’ viral kinetic response is a key determinant of treatment outcome. In particular achieving a rapid virological response (RVR) is the strongest of all predictors for SVR [8]. However, for some patients failure to do so does not preclude a successful SVR upon completion of therapy [8]. Identification of patients that are unlikely to respond to further treatment in the ‘RVR negative’ group would be of significant benefit to patients and clinicians enabling earlier treatment discontinuation and prioritisation for emerging therapies.

Discovery and validation of single nucleotide polymorphisms (SNPs) within the *IL28B* genetic region that strongly predict both spontaneous clearance and primary outcome to treatment has been a major breakthrough in the management of patients with Hepatitis C virus (HCV) infection [9]–[11]. We recently reported that the *IL28B* associated SNP, rs12979860, predicted spontaneous HCV viral clearance in a cohort of Irish patients mono-infected with HCV from contaminated anti-D blood products [12]. We found that the *IL28B-T* allele had a dominant effect in predicting the development of chronic HCV infection as the presence of an *IL28B-T* allele, either on its own or in a heterozygous state, was sufficient to confer an increased risk. We also identified a second genetic locus, the NK cell gene *KIR2DS3*, which predicted an increased risk of chronic HCV infection. There is substantial evidence emerging to support a role for NK cells during HCV infection [13]–[15]. The presence of these two risk factors synergised to increase the risk of developing chronic HCV infection over either risk factor alone. In the current study, we hypothesised that variation in *IL28B* and *KIR2DS3* genetic loci could improve identification of patients that achieve SVR in HCV/HIV-1 co-infected patients and furthermore, could potentially be used to identify those patients that despite not achieving RVR will ultimately go on to achieve an SVR.

## Materials and Methods

### Patients

Fully informed written consent was obtained from all participants and the study was granted local ethical approval from St. James’s Hospital Research Ethical Committee (Dublin) and the Ethics Committee of the Medical University of Vienna (Vienna). The study population comprised 149 HCV/HIV-1 co-infected patients attending specialist HCV/HIV co-infection clinics between June 2001 and December 2010, 71 in Dublin, Ireland and 78 in Vienna, Austria. Patient characteristics are shown in Table 1. HCV/HIV-1 co-infected patients were treated with pegylated-interferon alpha 2b 1.5 µg/kg/week (Peg-Intron®; Schering-Plough, Kenilworth, NJ, USA) or pegylated-interferon 2a 180 µg/week (Pegasys®; Roche, Basel, Switzerland) subcutaneously, and weight-based ribavirin (1000–1200 mg/day). Patients with HCV genotype 2/3 infection received treatment for either 24 weeks (Ireland) or 48 weeks (Austria), while those with genotype 1/4 infection received treatment for 48 weeks when HCV RNA was negative at week 24 of therapy. The *IL28B* associated SNP data for the Austrian patients has previously been published [16]. All patients were included for RVR analysis, with RVR defined as undetectable HCV RNA level by week four of treatment. Treatment success i.e. achievement of SVR was defined as undetectable plasma HCV RNA using the sensitive RT-PCR assay 24 weeks post-treatment cessation. Six patients were included in the analysis that achieved an SVR but did not complete the full treatment regimen. HCV genotype, HCV and HIV-1 viral load, CD4+ T cell count, and liver enzymes levels were recorded at baseline.

**Table 1:** Main clinical characteristics of the population included in on-treatment analysis.

	No SVR (n = 48)	SVR (n = 101)	P value
**Gender, M/F**	35 (0.729)/13 (0.271)	73 (0.77)/22 (0.23)	**NS, P>0.05**
**Age, years**	43.99±0.86	45.40±1.36	**NS, P>0.05**
**HCV Genotype**			
1	31 (0.65)	38 (0.37)	
2	0 (0.00)	8 (0.08)	
3	10 (0.21)	49 (0.49)	
4	7 (0.15)	5 (0.05)	
6	0 (0.00)	1 (0.01)	
**HCV Genotype 1/4 versus non 1/4**	38 (0.792)/10 (0.208)	43 (0.426)/58 (0.574)	**P = 0.0000278**
**HCV VL pre Rx, IU/ml (mean ± SEM)**	6433000±1221000	4965000±664700	NS, P>0.05
**ALT, U/ml (mean ± SEM)**	103.8±9.4	77.2±7.2	NS, P>0.05
**AST,U/ml (mean ± SEM)**	67±4.85	61±4.4	NS, P>0.05
**CD4^+^ pre Rx, cell counts/ml (mean ± SEM)**	509±21.2	510±27.7	NS, P>0.05
**HIV VL pre Rx, IU/ml (mean ± SEM)**	13400±11380	9830±2062	NS, P>0.05
**HAART, yes/no**	39 (0.81)/9 (0.18)	59 (0.62)/36 (0.38)	**P = 0.0205**

Patients who completed a course of pegylated-IFN and Ribavirin therapy were included in the SVR analysis. SVR, sustained virological response; VL, viral load; Rx, treatment; ALT, alanine aminotransferase; AST, aspartate aminotransferase; HAART, highly active antiretroviral therapy; NS, not significant.

### Genotyping for *KIR 2DS3* and the Associated SNP, rs12979860

Blood was collected in EDTA tubes following standard phlebotomy. DNA was isolated from whole blood, using the Qiamp DNA blood Mini Kit system (Qiagen, Hilden, Germany). The presence or absence of the *KIR2DS3* gene was determined using a PCR with sequence specific primers (PCR-SSP) method as described by Vilches et al. [17]. Genotyping for the rs12979860 SNP was performed using the ABI Taqman allelic discrimination kit [12], [18]. For routine quality control purposes, approximately 10% of samples were retyped anonymously, and no mismatches were found.

### Statistical Analysis

Continuous variables were presented as mean (± standard error of the mean) unless otherwise stated. Differences between continuous variables were evaluated by means of student’s t-test. Categorical baseline characteristics, genotype, allele and carrier frequency differences and odds ratio trend tests between populations were tested for significance by direct counting using a chi-square (**χ** ^2^) by EPI-INFO 3.5.1. Odds ratios (OR) were calculated and 95% confidence intervals are shown. P values less than 0.05 were considered significant. Statistical interaction between *KIR2DS3* and *IL28B-T* was evaluated by multinomial logistic regression (release 16.0; SPSS Inc.). Multinomial logistic regression is an extension of binary logistic regression that allows for more than two categories of the dependent or outcome variable. Baseline characteristics with a P<0.05 in univariate analysis were included as co-variates in multinomial logistic regression analysis and an adjusted odds ratio was generated.

## Results

### The *IL28B* SNP, rs12979860, is Associated wtih HCV Treatment Response in a Cohort of HCV/HIV-1 Co-infected Patients

The *IL28B* associated SNP, rs12979860, predicts both spontaneous and treatment outcomes to HCV infection [9], [12], [19], [20]. The *IL28B-C* allele is associated with HCV clearance while the *IL28B-T* allele is associated with a failure to do so. In order to test if the *IL28B* SNP, rs12979860, predicts responsiveness to HCV therapy in a HCV/HIV-1 co-infected setting, we typed a cohort of patients who had completed PEG-IFN and ribavirin treatment, for their *IL28B* genotype. Data on a subset of these patients has previously been published [16]. In the current cohort, sixty five patients were homozygous for the *IL28B-C* allele (CC, 0.436), 70 were heterozygous for *IL28B-CT* (CT, 0.470), and 14 patients were homozygous for *IL28B-T* allele (TT, 0.094). These genotypes were in Hardy-Weinberg equilibrium as expected.

Patients were stratified according to whether they achieved SVR following treatment and clinical characteristics of the groups are shown in Table 1. Baseline HCV genotype and HAART were significantly different between patients with SVR versus those that did not achieve SVR. In terms of *IL28B* analysis, *IL28B-CC* genotype was found much more frequently among patients with SVR (0.564, n = 57) compared to patients without SVR (0.167, n = 8; see Table 2). Conversely, the presence of the *IL28B-T* allele (*IL28B-CT* or *IL28B-TT*) was significantly over-represented in those patients that did not respond to treatment (0.833, n = 40) compared to those that cleared the virus (0.377, n = 44, P<0.000005). Thus, similar to what we have previously reported in spontaneous clearance [12], [18] and confirming what others have previously reported in HCV treatment response in a subset of this current cohort [18] the *IL28B* SNP, rs12979860, impacts HCV treatment responsiveness in patients co-infected with HCV and HIV-1.

**Table 2:** The *IL28B* SNP, rs12979860, is associated with HCV treatment response in a cohort of HCV/HIV-1 co-infected patients.

	Frequencies		No SVR vs SVR	
*IL28B* Genotype^1^	No SVR (n = 48)	SVR (n = 101)	χ^2^ (P)	OR (trend test)
CC	0.167 (8)	0.564 (57)	**20.396 (<0.00001)**	**1**
CT	0.666 (32)	0.377 (38)		**6.11**
TT	0.167 (8)	0.059 (6)		**9.67**

Patients that completed therapy were genotyped for the *IL28B* SNP (rs12979860) using a Taqman allele discrimination assay. Genotype frequencies were compared between patients who achieved SVR following treatment (n = 101) and those who did not achieve SVR (n = 48). Differences in frequency distribution between groups were tested for significance by a trend test.

1 Genotype for a subset of these patients, n = 72, has previously been described (Reference 16)

### *KIR2DS3* Gene Frequency is Increased in Co-infected Patients that Fail to Achieve SVR

As we had previously found that *KIR2DS3* was a genetic locus that predicted increased risk of developing chronic HCV infection, we hypothesised that it may also impact patient responsiveness to HCV treatment. Indeed, this was the case as the frequency of the *KIR2DS3* gene was significantly increased in patients that did not achieve SVR following treatment (0.438, n = 21) compared to those who did (0.218, n = 22, P<0.001; OR 2.79; 95% CI 1.25, 6.27, see Table 3). The carrier frequency of the *KIR2DS3* gene in the HCV/HIV-1 co-infected patients (0.289, n = 149), was similar to that found in a healthy, normal population (0.221, n = 136) [21].

**Table 3:** *KIR2DS3* gene frequency is increased in co-infected patients that fail to achieve SVR.

	Frequencies		No SVR vs SVR	
*KIR2DS3*	No SVR(n = 48)	SVR(n = 101)	χ^2^ (P)	OR (CI)
**Positive**	0.438 (21)	0.218 (22)	**7.65 (<0.01)**	**2.79 (1.75, 6.27)**
**Negative**	0.562 (27)	0.782 (79)		

*KIR2DS3* was genotyped in our cohort by PCR-SSP. The carrier frequency of *KIR2DS3* in 149 HCV/HIV-1 co-infected treated with peg-IFN and ribavirin was compared in 101 patients who achieved SVR following treatment and 48 patients who did not achieve SVR. Differences in frequency populations were tested for significance by χ^2^ test. Odds ratio with 95% confidence intervals is shown.

### The Presence of Both *IL28B*-*T* Allele and *KIR2DS3* Genetic Loci Significantly Increased the Risk of HCV Treatment Failure

**Figure 1:**
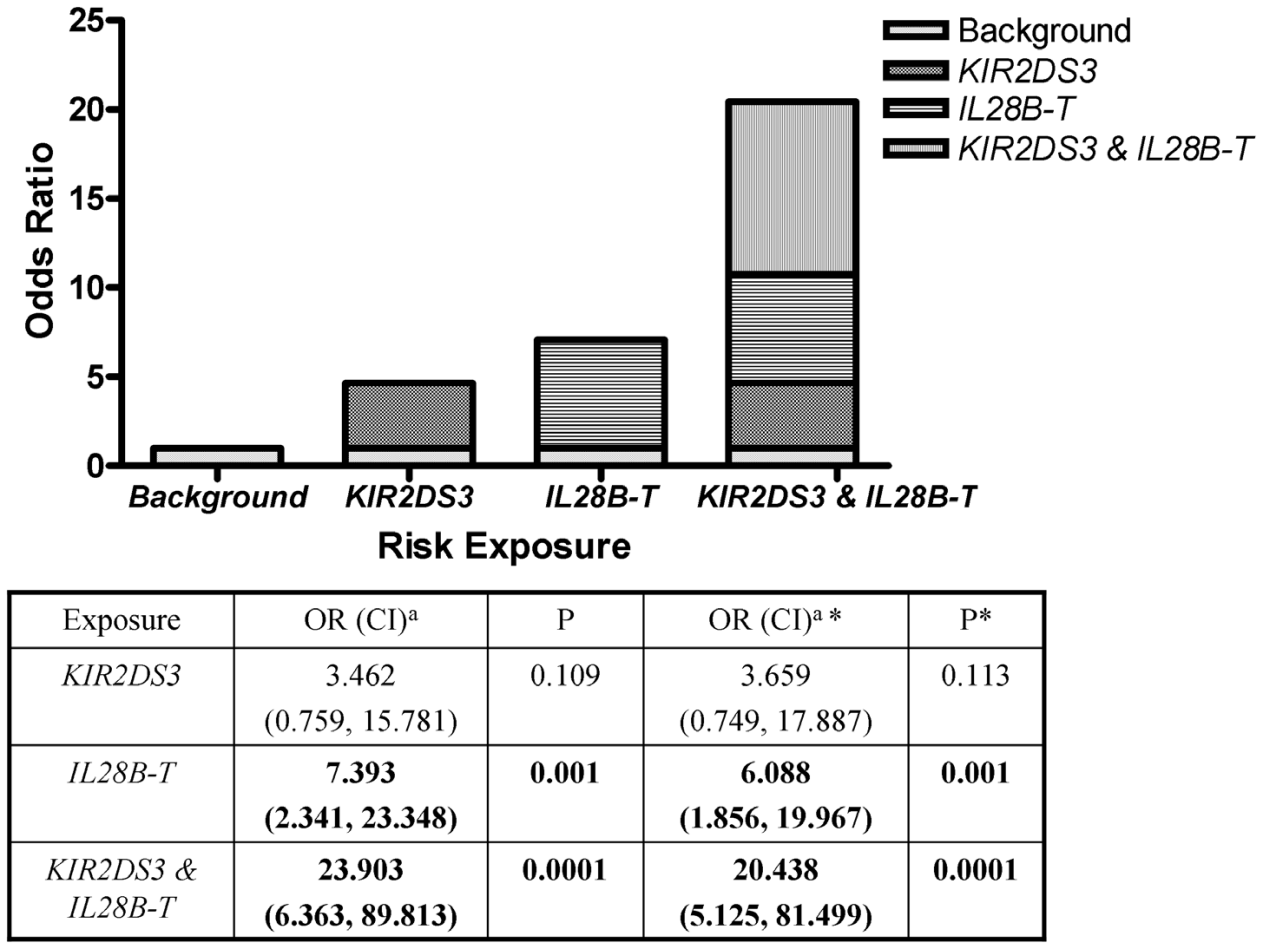
The presence of both *KIR2DS3* and *IL28B-T* alleles synergise to increase the odds of HCV treatment failure. ORs for *KIR2DS3* alone, *IL28B-T* carriers (CT or TT) alone, and *KIR2DS3* with *IL28B-T* compared with neither risk factor present were calculated from multinominal logisitic regression. The contribution of individual and combined risk factors to the ORs is graphically represented in the bar chart. Positive OR indicates an association with increased odds of treatment failure. ^a^Odds ratio (95% confidence interval). * adjusted OR including HCV genotype and HAART as co-variates.

Having identified two host genetic risk factors that modify treatment responsiveness in patients co-infected with HIV-1 and HCV, we examined if the presence of both risk factors influenced treatment outcomes compared to patients presenting with either one or no risk factor. When stratified, there was a significant difference between the groups (P<0.0005, OR 4.44, 95% CI 1.81, 10.96, see Table 4) indicating increased odds of treatment failure in patients having both *IL28B-T* and *KIR2DS3*. We therefore tested for potential interaction between these two risk factors. Using logistic regression to assess the independent predictive role of the two genetic loci, the presence of *KIR2DS3* became non-significant (OR 3.46, 95% CI 0.759, 15.781, P = 0.109) but the presence of *IL28B-T* and the presence of both alleles together (i.e., *KIR2DS3* carriers and *IL28B-T* carriers) remained significant. The presence of *KIR2DS3* along with *IL28-T* was associated with a strikingly increased OR in co-infected patients who failed to achieve SVR (Figure 1; OR 23.903, 95% CI 6.363, 89.813, P<0.0001). As HCV genotype and HAART were signifiantly different in univariate analysis, they were included as co-variates in multivariate logistic regression analysis. While HAART lost statistical significance, HCV genotype remained an independent predictor of treatment outcome. Adjusted OR for *IL28B* and *KIR2DS3* are shown in Figure 1. In summary the presence of both risk alleles, *KIR2DS3* and *IL28B-T*, synergised to significantly increase the odds of failing to achieving SVR compared to the presence of either marker alone.

**Table 4:** The presence of both *IL28B*-*T* and *KIR2DS3* significantly increased the odds of HCV treatment failure.

Genotype	No SVR (n = 48)	SVR (n = 101)	χ^2^(P)	OR (CI )
**2DS3+T+**	0.396 (19)	0.129 (13)	**13.77 (<0.0005)**	**4.44 (1.81, 10.96)**
**Presence of none** **or one risk factor**	0.604 (29)	0.827 (88)		

Patients were stratified according to the carriage of both risk factors (*IL28B-T* allele and KIR gene *2DS3*) compared to either no risk factor or just one present. The frequency of the genotypes between patients that achieved SVR (n = 101) was compared with those that failed to clear virus (n = 48) and tested for significance by χ^2^ test.

### *IL28B-T* and *KIR2DS3* Identify RVR Negative Patients who Ultimately Fail to Achieve SVR

We investigated if *IL28B-T* or *KIR2DS3* affected RVR in our cohort and found that while *KIR2DS3* did not, carriage of *IL28B-T* was associated with failure to achieve RVR in our patients (Table 5).

**Table 5:** The *IL28B* SNP, rs12979860, is associated with RVR in a cohort of HCV/HIV-1 co-infected patients.

	Frequencies		No RVR vs RVR	
*IL28B* Genotype	No RVR (n = 88)	RVR (n = 61)	χ^2^ (P)	OR (trend test)
***CC***	0.296 (28)	0.607 (37)	**9.807 (<0.005)**	**1**
***CT***	0.625 (50)	0.328 (20)		**3.30**
***TT***	0.114 (10)	0.066 (4)		**3.30**

Patients were genotyped for the *IL28B*-associated SNP, rs12979860. Frequencies of the various genotypes were compared between patients that achieved an RVR (n = 61) and those that did not achieve RVR (n = 88) after 4 weeks of treatment. A χ^2^ trend test was used to compare the data.

Most patients who achieved RVR and completed a full course of HCV therapy achieved SVR (93%; 57/61 such patients in this current cohort). In our cohort, 50% of patients that did not achieve RVR finally achieved SVR while 50% failed to do so. We considered whether *IL28B-T* or *KIR2DS3* was associated with responsiveness to treatment in these HCV/HIV-1 co-infected patients without RVR. RVR negative patients (n = 88) were stratified into four possible “risk genotypes”, according to the presence and/or absence of *IL28B-T* and *KIR2DS3* (see Table 6). Among 20 patients with no host genetic risk factors (i.e. *IL28B-CC* and *KIR2DS3* negative) 18 (90%) cleared virus despite not achieving an RVR. The presence of a single risk factor (either *IL28B-T* or *KIR2DS3*) could not sufficiently identify patients likely to achieve SVR in the RVR negative patient group. However, the presence of two host genetic risk factors was strongly associated with treatment failure as only 24% of this group achieved SVR. Thus, host genetic risk factors can increase the potential to identify RVR negative patients that are highly unlikely to respond to therapy.

**Table 6:** Presence of host genetic risk factors identifies RVR negative patients that will fail to achieve SVR on completion of therapy.

Genotype								
*IL28B/2DS3*	*IL28B-CC/2DS3+*		*IL28B-CC/2DS3-*		*IL28B-T/2DS3+*		*IL28B-T/2DS3-*	
**Risk factors**	1		0		**2**		1	
**Treatment outcome**	**SVR**	**No** **SVR**	**SVR**	**No** **SVR**	**SVR**	**No** **SVR**	**SVR**	**No** **SVR**
**Counts (n = 88)**	4	4	18	2	**5**	**16**	17	22
**Treatment**	Continue		Continue		**Stop**		Continue	

Patients that failed to achieve RVR after 4 weeks of treatment were analysed. Four different host genetic genotypes were identified based on the presence of 2 risk factors (*IL28B-T* allele, and presence of the NK cell gene *KIR2DS3*). Patients were stratified according to whether they ultimately achieved SVR or not within each genotype group and the counts obtained are shown. Based on these data, a suggested treatment strategy is shown with respect to supporting patients through treatment or terminating treatment early.

## Discussion

We have recently identified an *IL28B* SNP (rs12979860) and a second immune gene, *KIR2DS3*, that function as host risk factors predicting failure to spontaneously clear HCV virus by immune mediated mechanisms [12]. In the present study, we investigated if these two host genetic risk factors could also impact patient response to HCV treatment in patients that are co-infected with HIV-1. The *IL28B*-associated SNP was associated with increased odds of treatment failure in our patients. This supports previously reported data for this SNP in which *IL28B-CC* was a strong predictor of SVR in HIV-1/HCV co-infected patients [16], [22], [23]. The relative contribution of *IL28B* major and minor alleles to treatment outcome is not yet clear, although a dominant effect of the *IL28B-T* allele has been reported for spontaneous and treatment induced viral clearance [9], [12]. Our data suggest that carriage of the *IL28B-C* allele in co-infected patients conferred a benefit while carriage of *IL28B-T* allele conferred a risk to the host in terms of treatment response. Understanding the molecular mechanisms involved to explain these findings is a significant challenge as the biological effects of *IL28B* associated SNPs have not yet been elucidated [24].

We also found that *KIR2DS3*, an NK cell associated gene, increased the odds of failing to achieve SVR in response to treatment in HCV/HIV-1 infected individuals. There is currently no known function for *KIR2DS3* [16] and evidence suggests that it is not expressed at the cell surface [25]. It is likely that *KIR2DS3* serves as a genetic marker for a closely related tightly linked KIR gene that mediates the biological effect. Although a mechanism is unclear, presence of *KIR2DS3* predicted increased odds of treatment failure in our patient cohort. However, when stratified for independence *KIR2DS3* does not appear to be have a role independent of *IL28B-T* but rather increases its negative effect on treatment outcome. We found the presence of both risk loci, *KIR2DS3* and *IL28B-T*, significantly increases the odds of failing to achieve SVR when compared to the presence of either marker alone and this improves identification of patients with a high likelihood of SVR prior to commencing antiviral therapy. Combining these newly identified host genetic factors with other recognised predictors of SVR (age, baseline HCV-RNA levels, HCV genotype, liver fibrosis stage, pre-treatment IP10 levels) will aid patient management by improving prediction potential of SVR prior to embarking on PEG-IFN and ribavirin treatment. Furthermore, we have confirmed a genetic synergy between *IL28B* and *KIR2DS3* in HCV viral clearance, originally observed in spontaneous clearance and now defined in the treatment induced setting. Although a mechanism for the synergy is not yet known, it is clear that for patients with genotype 1/4 HCV and with both host genetic risk factors, an early and sustained impaired ability to clear virus in response to treatment is evident (see Figure S1). There was no observable impact of host immune genotype on viral kinetics for patients with non-genotype 1/4 HCV that may reflect their well documented rapid response to treatment. These data support the growing body of evidence that the innate immune system plays an important role in HCV clearance.

Achievement of RVR is highly predictive of SVR upon completion of a full duration of PEG-IFN and ribavirin. However, failure to achieve RVR in isolation does not sufficiently discriminate responders from non-responders (50% of RVR negative group achieved SVR in this study) and therefore treatment is continued in all individuals. Identification of factors that predict SVR in these RVR negative patients would significantly improve clinical management of this patient subgroup, and the data presented here provide an important contribution towards this goal. Combining *IL28B* and *KIR2DS3* genotypes increased the likelihood of failing to achieve SVR in the RVR negative cohort of patients. This effect appeared to be independent of HCV genotype although sample numbers were small upon stratification (data not shown). In the RVR negative patients the negative predictive value (indicating correct prediction of treatment failure) of using both risk genetic loci increases to 95.45% (with sensitivity of 90% and specificity of 61,76%) compared to 86.36% (with sensitivity of 78.57% and specificity of 64.41%) for the presence of *IL28B-T* alone. These data suggest that patients that have achieved a RVR, or have one or no genetic risk factors should continue standard treatment. However, co-infected patients with unfavourable pre-treatment characteristics (i.e. HCV genotype 1/4 and/or presence of at least two host genetic risk factors, see Table 6) who fail to achieve RVR are highly unlikely to achieve SVR. For these patients, where primary outcome of treatment is to obtain SVR and not just secondary gain of histological stability, therapy could be stopped. Individualising care pathways would limit toxicity in those unlikely to respond and lower costs associated with ineffective treatment. These patients could then be prioritised for access to newer non-IFN based direct acting antiviral agents as they become approved in co-infected patients. While the data need to be validated in additional cohorts, the results strongly suggest that host genetic analysis needs to become a routine consideration during management of care pathways in HCV/HIV-1 co-infection.

## Supporting Information

Figure S1 On treatment HCV kinetic response stratified according to innate immune gene risk factors. Kinetic responses are shown for patients with HCV genotypes 1/4 (A) and non-1/4 genotypes (B). Black squares indicate carriage of 1 or no innate immune gene risk factors; Black triangle, carriage of both *IL28B-T* and *KIR2DS3*. Symbols shown mean value and bars show standard error of the mean (SEM).
(TIFF)

## References

[1] Kim AY, Chung RT (2009) Coinfection with HIV-1 and HCV–a one-two punch. Gastroenterology 137: 795–814. 19549523 10.1053/j.gastro.2009.06.040 PMC3146750

[2] Vogel M, Page E, Boesecke C, Reiberger T, Schwarze-Zander C, et al (2012) Liver fibrosis progression after acute hepatitis C virus infection in HIV-positive individuals. Clin Infect Dis 54: 556–559. 22156856 10.1093/cid/cir854

[3] Operskalski EA, Kovacs A (2011) HIV/HCV co-infection: pathogenesis, clinical complications, treatment, and new therapeutic technologies. Curr HIV/AIDS Rep 8: 12–22. 21221855 10.1007/s11904-010-0071-3 PMC3035774

[4] Bacon BR, Gordon SC, Lawitz E, Marcellin P, Vierling JM, et al (2011) Boceprevir for previously treated chronic HCV genotype 1 infection. N Engl J Med 364: 1207–1217. 21449784 10.1056/NEJMoa1009482 PMC3153125

[5] Poordad F, McCone J Jr, Bacon BR, Bruno S, Manns MP, et al (2011) Boceprevir for untreated chronic HCV genotype 1 infection. N Engl J Med 364: 1195–1206. 21449783 10.1056/NEJMoa1010494 PMC3766849

[6] Jacobson IM, McHutchison JG, Dusheiko G, Di Bisceglie AM, Reddy KR, et al (2011) Telaprevir for previously untreated chronic hepatitis C virus infection. N Engl J Med 364: 2405–2416. 21696307 10.1056/NEJMoa1012912

[7] Thomas DL, Bartlett JG, Peters MG, Sherman KE, Sulkowski MS, et al (2012) Provisional guidance on the use of hepatitis C virus protease inhibitors for treatment of hepatitis C in HIV-infected persons. Clin Infect Dis 54: 979–983. 22173234 10.1093/cid/cir882 PMC3404695

[8] Shea DO, Tuite H, Farrell G, Codd M, Mulcahy F, et al (2008) Role of rapid virological response in prediction of sustained virological response to Peg-IFN plus ribavirin in HCV/HIV co-infected individuals. J Viral Hepat 15: 482–489. 18221297 10.1111/j.1365-2893.2008.00969.x

[9] Ge D, Fellay J, Thompson AJ, Simon JS, Shianna KV, et al (2009) Genetic variation in IL28B predicts hepatitis C treatment-induced viral clearance. Nature 461: 399–401. 19684573 10.1038/nature08309

[10] Suppiah V, Moldovan M, Ahlenstiel G, Berg T, Weltman M, et al (2009) IL28B is associated with response to chronic hepatitis C interferon-alpha and ribavirin therapy. Nat Genet 41: 1100–1104. 19749758 10.1038/ng.447

[11] Tanaka Y, Nishida N, Sugiyama M, Kurosaki M, Matsuura K, et al (2009) Genome-wide association of IL28B with response to pegylated interferon-alpha and ribavirin therapy for chronic hepatitis C. Nat Genet. 41: 1105–1109. 10.1038/ng.449 19749757

[12] Dring MM, Morrison MH, McSharry BP, Guinan KJ, Hagan R, et al (2011) Innate immune genes synergize to predict increased risk of chronic disease in hepatitis C virus infection. Proc Natl Acad Sci U S A 108: 5736–5741. 21402922 10.1073/pnas.1016358108 PMC3078345

[13] Khakoo SI, Thio CL, Martin MP, Brooks CR, Gao X, et al (2004) HLA and NK cell inhibitory receptor genes in resolving hepatitis C virus infection. Science 305: 872–874. 15297676 10.1126/science.1097670

[14] Lodoen MB, Lanier LL (2006) Natural killer cells as an initial defense against pathogens. Curr Opin Immunol 18: 391–398. 16765573 10.1016/j.coi.2006.05.002 PMC7127478

[15] Ahlenstiel G, Titerence RH, Koh C, Edlich B, Feld JJ, et al.. (2010) Natural killer cells are polarized toward cytotoxicity in chronic hepatitis C in an interferon-alfa-dependent manner. Gastroenterology 138: 325–335 e321–322. 10.1053/j.gastro.2009.08.066 PMC2862622 19747917

[16] Payer BA, Reiberger T, Aberle J, Ferenci P, Holzmann H, et al (2012) IL28B and interferon-gamma inducible protein 10 for prediction of rapid virologic response and sustained virologic response in HIV-HCV-coinfected patients. Eur J Clin Invest 42: 599–606. 22117591 10.1111/j.1365-2362.2011.02623.x

[17] Vilches C, Castano J, Gomez-Lozano N, Estefania E (2007) Facilitation of KIR genotyping by a PCR-SSP method that amplifies short DNA fragments. Tissue Antigens 70: 415–422. 17854430 10.1111/j.1399-0039.2007.00923.x

[18] Beinhardt S, Aberle JH, Strasser M, Dulic-Lakovic E, Maieron A, et al.. (2012) Serum level of IP-10 increases predictive value of IL28B polymorphisms for spontaneous clearance of acute HCV infection. Gastroenterology 142: 78–85 e72. 10.1053/j.gastro.2011.09.039 22192885

[19] Asselah T (2010) Genetic polymorphism and response to treatment in chronic hepatitis C: the future of personalized medicine. J Hepatol 52: 452–454. 20133003 10.1016/j.jhep.2009.11.016

[20] Fellay J, Ge D, Shianna KV, Colombo S, Ledergerber B, et al (2009) Common genetic variation and the control of HIV-1 in humans. PLoS Genet 5: e1000791. 20041166 10.1371/journal.pgen.1000791 PMC2791220

[21] Guinan KJ, Cunningham RT, Meenagh A, Gonzalez A, Dring MM, et al (2010) Signatures of natural selection and coevolution between killer cell immunoglobulin-like receptors (KIR) and HLA class I genes. Genes Immun 11: 467–478. 20200544 10.1038/gene.2010.9

[22] Pineda JA, Caruz A, Rivero A, Neukam K, Salas I, et al (2010) Prediction of response to pegylated interferon plus ribavirin by IL28B gene variation in patients coinfected with HIV and hepatitis C virus. Clin Infect Dis 51: 788–795. 20804372 10.1086/656235

[23] Rallon NI, Naggie S, Benito JM, Medrano J, Restrepo C, et al (2010) Association of a single nucleotide polymorphism near the interleukin-28B gene with response to hepatitis C therapy in HIV/hepatitis C virus-coinfected patients. AIDS 24: F23–29. 20389235 10.1097/QAD.0b013e3283391d6d PMC4892373

[24] Wapner J (2010) Pharmacogenomics. Gene variants affect hepatitis C treatment, but link is elusive. Science 330: 579. 21030622 10.1126/science.330.6004.579

[25] VandenBussche CJ, Mulrooney TJ, Frazier WR, Dakshanamurthy S, Hurley CK (2009) Dramatically reduced surface expression of NK cell receptor KIR2DS3 is attributed to multiple residues throughout the molecule. Genes Immun 10: 162–173. 19005473 10.1038/gene.2008.91 PMC3487464

